# Marked effects of intracranial volume correction methods on sex differences in neuroanatomical structures: a HUNT MRI study

**DOI:** 10.3389/fnins.2015.00238

**Published:** 2015-07-09

**Authors:** Carl W. S. Pintzka, Tor I. Hansen, Hallvard R. Evensmoen, Asta K. Håberg

**Affiliations:** ^1^Department of Neuroscience, Norwegian University of Science and TechnologyTrondheim, Norway; ^2^Department of Medical Imaging, St. Olav's University HospitalTrondheim, Norway

**Keywords:** brain, sexual dimorphism, magnetic resonance imaging, head size correction, proportion

## Abstract

To date, there is no consensus whether sexual dimorphism in the size of neuroanatomical structures exists, or if such differences are caused by choice of intracranial volume (ICV) correction method. When investigating volume differences in neuroanatomical structures, corrections for variation in ICV are used. Commonly applied methods are the ICV-proportions, ICV-residuals and ICV as a covariate of no interest, ANCOVA. However, these different methods give contradictory results with regard to presence of sex differences. Our aims were to investigate presence of sexual dimorphism in 18 neuroanatomical volumes unrelated to ICV-differences by using a large ICV-matched subsample of 304 men and women from the HUNT-MRI general population study, and further to demonstrate in the entire sample of 966 healthy subjects, which of the ICV-correction methods gave results similar to the ICV-matched subsample. In addition, sex-specific subsamples were created to investigate whether differences were an effect of head size or sex. Most sex differences were related to volume scaling with ICV, independent of sex. Sex differences were detected in a few structures; amygdala, cerebellar cortex, and 3rd ventricle were larger in men, but the effect sizes were small. The residuals and ANCOVA methods were most effective at removing the effects of ICV. The proportions method suffered from systematic errors due to lack of proportionality between ICV and neuroanatomical volumes, leading to systematic mis-assignment of structures as either larger or smaller than their actual size. Adding additional sexual dimorphic covariates to the ANCOVA gave opposite results of those obtained in the ICV-matched subsample or with the residuals method. The findings in the current study explain some of the considerable variation in the literature on sexual dimorphisms in neuroanatomical volumes. In conclusion, sex plays a minor role for neuroanatomical volume differences; most differences are related to ICV.

## Introduction

There are well-known sex differences in the prevalence, age-of-onset and severity of several brain-related diseases, including Alzheimer's disease, stroke, multiple sclerosis, bipolar- and autistic disorder, depression, Parkinson's disease and schizophrenia (Iacono and Beiser, 1992; Hirtz et al., 2007; American Psychiatric Association, 2013). In such sexually dimorphic brain diseases, a potential sex-specific protective agent might exist that could lead to future therapies (Mccarthy et al., 2012). Investigating the impact of sex on brain structure is thus important to improve our understanding of both the normal brain and brain pathologies. When investigating sex differences in volume of neuroanatomical structures, it is customary to correct for variations in intracranial volume (ICV) since corrected neuroanatomical volumes are believed to be more valid than absolute volumes in describing structure-function relationships (Sanfilipo et al., 2004). There are several ways of estimating ICV. While manual delineation is considered the criterion standard, it is labor-intensive and automatic methods implemented in software like FreeSurfer and SPM are frequently used. These automated methods have been compared to manual delineation and found to have acceptable agreement (Keihaninejad et al., 2010; Hansen et al., 2015; Malone et al., 2015). The three most frequently used ICV-correction methods are the ICV-proportions, the ICV-residuals and the ICV as a covariate of no interest, ANCOVA, method (O'brien et al., 2011). In studies with two healthy groups, comparing for example men and women, the residuals method and the ANCOVA method with only ICV as covariate are identical (O'brien et al., 2011; Nordenskjöld et al., 2015).

To date, there is no consensus whether sexual dimorphism in the size of neuroanatomical structures exists, or if such differences are caused by differences in ICV correction methods between the studies. Table 1 highlights the conflicting results reported in the literature concerning sexual dimorphism in the size of neuroanatomical structures. Methodologically these studies vary significantly in regard to sample characteristics (e.g., age, number, and ethnicity of the subjects), MRI hardware (e.g., field strength, coils), and scan protocols (e.g., resolution and contrast), and image analysis approach (e.g., manual/automated ICV and neuroanatomical volume segmentation). The importance of ICV-correction method may be particularly relevant for the lack of consensus with regard to sexual dimorphism in neuroanatomical volumes. Indeed, studies that use the proportions method generally find larger gray matter structures in women (Filipek et al., 1994; Goldstein et al., 2001; Szabó et al., 2003; Kruggel, 2006; Chen et al., 2007; Leonard et al., 2008; Inano et al., 2013; Perlaki et al., 2014; Voevodskaya et al., 2014) while studies using the residuals or ANCOVA method generally report larger gray matter structures in men (Raz et al., 2001, 2004; Fjell et al., 2009) or no sex difference (Gur et al., 2002; Barnes et al., 2010; Perlaki et al., 2014; Voevodskaya et al., 2014) (Table 1). In the two largest studies that have investigated the effects of age and sex on volume of neuroanatomical structures, highly contradictory results were reported (Fjell et al., 2009; Inano et al., 2013). Fjell and colleagues investigated 1143 healthy subjects (18–94 years) with the residuals method whereas Inano and colleagues investigated 861 healthy subjects (24–84 years) using the proportions method. The two studies agreed on only 3 out of 17 structures (~18%), i.e., no sex difference in cerebral white matter and caudate volumes, plus larger 3rd ventricle volume in men. Other than choice of ICV-correction method these studies are very similar, pointing to a significant effect of ICV-correction method. Furthermore, the effects of sex on neuroanatomical volumes are generally found to be small (Fjell et al., 2009; Inano et al., 2013; Voevodskaya et al., 2014), thus large datasets are required to examine the relationships between ICV and sex on neuroanatomical volumes.

**Table 1:** **Comparison of sex differences across selected studies**.

	**ICV-correction method**	***N***	**Age range**	**Segmentation**	**GM**	**WM**	**CSF**	**Cerebellum**	**HC**	**CC**
Barnes et al., 2010	ANCOVA	78	24–81	Freesurfer	NS	↑F	NA	NA	NS	NA
Chen et al., 2007	ANCOVA	411	44–48	SPM	NS	NS	NS	NA	NA	NA
Gur et al., 2002	ANCOVA	116	18–49		↑F^a^	NS	NS	NS	NS	NA
Perlaki et al., 2014	ANCOVA	99	19–31	Freesurfer	NA	NA	NA	NA	NS	NA
Raz et al., 2001	ANCOVA	190	18–81	Manual	NA	NA	NA	↑M	NA	NA
Blatter et al., 1995	Proportions	194	16–65	ANALYZE	NS	NS	NS	NA	NA	NA
Chen et al., 2007	Proportions	411	44–48	SPM	↑F	↑M	↑M	NA	NA	NA
Filipek et al., 1994	Proportions	20	17–37	Manual	NS	NS	NS	↑F	NA	NA
Goldstein et al., 2001	Proportions	48	~39	Manual	↑F	NS	↑M	NA	NS	NA
Gur et al., 1999	Proportions	80	18–45		↑F	↑M	↑M	NA	NA	NA
Inano et al., 2013	Proportions	861	24–84	Freesurfer	↑F	NS	↑M	↑F^b^	↑F	NA
Kruggel, 2006	Proportions	502	16–70		↑F	↑M	↑F	NS	NA	NA
Leonard et al., 2008	Proportions	200	~21	FSL	↑F	↑M	NS	↑F	NA	↑F
Mu et al., 1999	Proportions	619	40–90	Manual	NA	NA	NA	NA	NS	NA
Perlaki et al., 2014	Proportions	99	19–31	Freesurfer	NA	NA	NA	NA	↑F	NA
Sullivan et al., 2001	Proportions	92	22–71	Manual	NA	NA	NA	NA	NA	NS
Szabó et al., 2003	Proportions	34	19–38	Manual	NA	NA	NA	NS	↑F	NA
Voevodskaya et al., 2014	Proportions	406	75	Freesurfer	NA	NA	↑M	↑F	↑F	↑F
Xu et al., 2000	Proportions	331	30–79	Manual	NA	NA	NA	↑M	NA	NA
Fjell et al., 2009	Residuals	1143	18–94	Freesurfer	↑M	NS	NS^c^	↑M^d^	↑M	NA
Raz et al., 2004	Residuals	200	~47	Manual	↑M	↑M	NA	NA	↑M	NA
Sullivan et al., 2001	Residuals + ANCOVA	92	22–71	Manual	NA	NA	NA	NA	NA	↑M
Voevodskaya et al., 2014	Residuals	406	75	Freesurfer	NA	NA	NS	NS	NS	NS

*GM, gray matter (includes either total gray matter or total cerebral cortex volume); WM, white matter; CSF, cerebrospinal fluid (either reported as CSF or total ventricular volume); HC, hippocampus; CC, corpus callosum; NA, not applicable; NS, not significant; ↑F, larger volume in women; ↑M, larger volume in men*.

a *Only significant for the orbitofrontal cortex*.

b *Only significant for white matter*.

c *Significant for the 3rd ventricle (larger in men)*.

d *Only significant for gray matter*.

Previous studies comparing different ICV-correction methods generally recommend using the residuals or ANCOVA method (Arndt et al., 1991; Mathalon et al., 1993; Sullivan et al., 2001; Sanfilipo et al., 2004; O'brien et al., 2011; Perlaki et al., 2014; Nordenskjöld et al., 2015). These studies focused mainly on theoretical and reliability issues, used pediatric or geriatric samples, or only investigated a few structures. The practical implications of the different ICV-correction methods have received limited attention and there is no consensus how to adjust for ICV. Recently, one study investigated the effects of different ICV-correction methods in a cohort of 406 healthy 75 year old subjects (Voevodskaya et al., 2014). No sex differences in neuroanatomical volumes were found when using the residuals method, and large effects of different ICV-correction methods on neuroanatomical volumes were reported. The lack of sex differences detected when using the residuals method might be related to sample size, as sex effects are generally small. Moreover, the y-intercept of the regression line between neuroanatomical structure and ICV is of importance when interpreting the results of the proportions method, as described in Barnes et al. (2010) and Nordenskjöld et al. (2015). However, the role of the y-intercept in overestimating or underestimating the volume of neuronatomical structures has not been investigated in subcortical structures. Based on the discrepant results in Fjell et al. (2009) and Inano et al. (2013), combined with the possibility that y-intercept differences lead to systematic errors when examining group differences in neuroanatomical volumes, we decided to explore the relationship between ICV, sex, and neuroanatomical volumes in a large sample.

There were several aims for the current study. First, we wanted to investigate presence of sexual dimorphism in neuroanatomical volumes unrelated to ICV-differences by using a large ICV-matched subsample of 304 men and women, and thereby establishing the ground truth. Secondly, we wanted to investigate the relationship between ICV and the relative size of different brain tissues, expressed as proportions of ICV. Thirdly, we wanted to investigate how each ICV-correction method influenced presence of sex differences in neuroanatomical volumes, in particular, which method gave results similar to the ground truth obtained in the ICV-matched subsample, and moreover whether the differences were an effect of head size or sex. Finally, we wanted to investigate the extent including additional covariates changed the results compared to the results obtained without the additional covariates and the ICV-matched subsample. To this end, we studied sexual dimorphism in 18 neuroanatomical structures in a subsample of 304 men and women with matching ICVs from a general population (HUNT-MRI) study, and the impact of the different ICV-correction methods on sexual dimorphisms in the same structures in 966 healthy subjects from the HUNT-MRI study. Furthermore, sex-specific subsamples were created to investigate whether the results were an effect of head size or sex. The relationship between brain tissue type and ICV was investigated using stratified groups of increasing ICVs including both men and women. Finally, we investigated correlations between the results from the ICV-matched subsample and the different ICV-correction methods. To the best of our knowledge, no study has implemented one to one ICV-matching and different ICV-correction methods on a large dataset from a representative sample of a general population where participant and non-participant characteristics are described (Honningsvag et al., 2012) to investigate their impact on sexual dimorphisms in neuroanatomical volumes.

## Materials and methods

### The HUNT population and the HUNT-MRI cohort

The study was approved by the HUNT study board of directors and the regional ethics and health research committee (2011/456). All participants gave their informed written consent.

The Nord-Trøndelag Health Study (The HUNT Study) is a collaboration between HUNT Research Centre (Faculty of Medicine, Norwegian University of Science and Technology NTNU), Nord-Trøndelag County Council, Central Norway Health Authority, and the Norwegian Institute of Public Health (Krokstad et al., 2013). It is a large multiphase, multipurpose health study on the inhabitants = 13 years in the county of Nord-Trøndelag. The inhabitants of Nord-Trøndelag county have been invited to participate in three waves; HUNT1 (1984–86), HUNT2 (1995–97) and HUNT3 (2006–08). The overall participation rates were 89.4, 69.5, and 54.1%, respectively. However, in HUNT3 the participation rate for the age group 60–69 was 71.1%. A non-participation study from HUNT3 showed that non-participants had lower socioeconomic status, higher mortality and higher prevalence's of several chronic diseases, whilst opposite patterns were found for common problems like musculoskeletal pain, urine incontinence and headache (Langhammer et al., 2012). HUNT MRI was a substudy after HUNT3. The cohort invited to participate in HUNT MRI was drawn from the HUNT population, but limited to volunteers who had participated in HUNT 1, 2, and 3, and were between 50 and 65 years at time of inclusion in the HUNT MRI study. In total 73% of those invited to HUNT MRI agreed to participate (Honningsvag et al., 2012). The HUNT MRI cohort consisted of 1006 subjects (530 women). A study comparing participants and non-participants in the HUNT MRI study and subjects from the HUNT cohort not invited found that the groups were not widely different, but HUNT MRI participating women had a higher level of education, lower body mass index, lower blood pressure, but there was no difference with regard to number of individuals with hypertension, and fewer had fasting blood glucose ≥5.6 mmol/l, but no difference in number of diabetic individuals (Honningsvag et al., 2012).

### MRI scan protocol

All imaging was performed on the same 1.5 T General Electric Signa HDx 1.5 T MRI scanner equipped with an eight channel head coil (GE Healthcare) and software version pre-14.0M4. The examinations were conducted by eight MRI technologists following a standardized written and illustrated procedure. All volunteers underwent the same scan protocol. In the current study the Alzheimer's disease Neuroimaging Initiate (ADNI) volume, (http://adni.loni.usc.edu/methods/documents/mri-protocols/) which is a T1 weighted MPRAGE volume (TR = 10,156 ms, TE = 4.044 ms, FOV = 240 mm, slice thickness = 1.2 mm, gap 0 mm, matrix 192 × 192, giving an in plane resolution of 0.94 × 0.94 mm) was used, as well as an axial T2 weighted sequence used in the ICV estimation (TR = 7.84 ms, TE = 95 ms, FOV = 230 mm, slice thickness = 4 mm, gap 1 mm, matrix 512 × 320, giving an in plane resolution of 0.45 × 0.45 mm).

### Image processing

**Figure 1:**
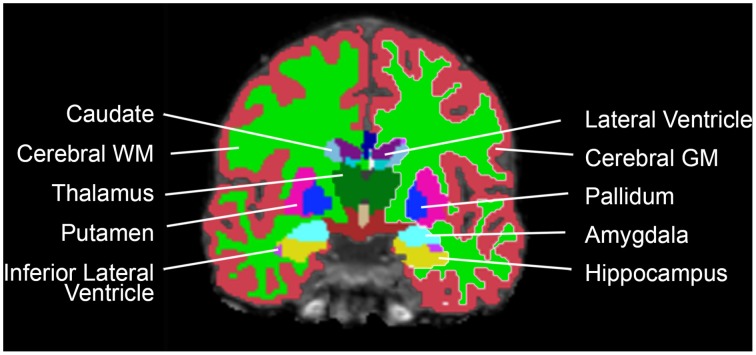
**Coronal view of one participant's brain, with the Freesurfer segmentations superimposed**.

The datasets were analyzed with FreeSurfer 4.50 (http://surfer.nmr.mgh.harvard.edu/) for automatic segmentation and SPM8 (rel. 5236) (www.fil.ion.ucl.ac.uk/spm) for ICV estimation. The volumes of 18 neuroanatomical structures (accumbens, amygdala, brainstem, caudate, cerebellar cortex, cerebellar white matter, cerebral cortex, cerebral white matter, cerebrospinal fluid (CSF), hippocampus, inferior lateral ventricle, lateral ventricle, pallidum, putamen, thalamus, total brain volume, 3rd ventricle, 4th ventricle) were segmented using an automated procedure described previously (Fischl et al., 2002) (Figure 1). In structures that come in pairs, the sum of left and right hemisphere was used. Cortical gray matter was defined as the volume of the cerebral and cerebellar cortex; white matter was defined as the volume of the cerebral and cerebellar white matter; subcortical gray matter was defined as the volume of the accumbens, amygdala, caudate, hippocampus, pallidum, putamen, and thalamus; the ventricles were defined as the volume of the 3rd and 4th ventricle, the inferior lateral and lateral ventricle and the CSF. To avoid bias, all Freesurfer results were visually inspected by a blinded colleague at the Multimodal imaging lab, UCSD, where the data were analyzed, and all subpar datasets removed. ICV was estimated using an automated version of the reverse brain mask method (RBM) (Keihaninejad et al., 2010) called the “automatic reverse brain mask method” by using the “new segment” approach of the SPM8 toolbox, full description in Hansen et al. (2015). This method was recently shown to have improved accuracy compared to the ICV estimate generated by Freesurfer (Hansen et al., 2015) and comparable to the SPM12 results obtained in Malone et al. (2015).

### ICV-matched subsample

A subsample of men and women with matching ICV was created by matching each subject with one from the opposite sex with an ICV≤ 10 ml different. A total of 152 pairs of ICV-matched men and women were found which resulted in a subsample of 304 subjects.

### Sex specific subsamples

**Figure 2:**
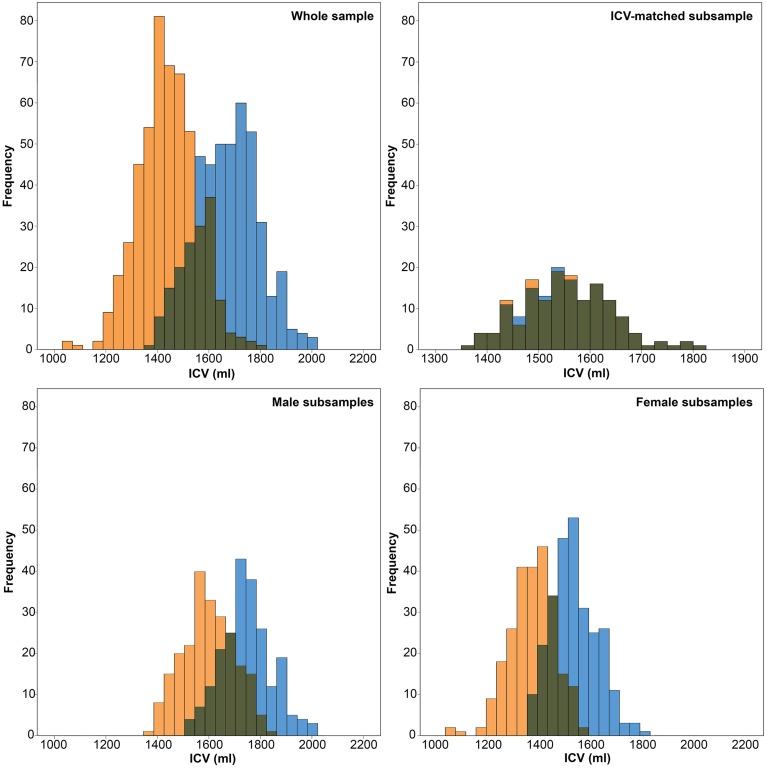
**Histograms of intracranial volumes (ICV). Top left:** The frequency of ICV in the whole sample for women (orange) and men (blue). **Top right**: The frequency of ICV in the ICV-matched subsample for women (orange) and men (blue). **Bottom left**: The frequency of ICV in the male subsample for the small-ICV group (orange) and large-ICV group (blue). **Bottom right**: The frequency of ICV in the female subsample for the small-ICV group (orange) and large-ICV group (blue).

To directly test the effect of ICV on neuroanatomical volumes, and to investigate whether ICV or sex mediates most differences, the total sample of 966 HUNT MRI participants was dichotomized into sex-specific subsamples; one men- and one women-only subsample. Subsequently, each sex specific subsample was subdivided into two groups using probability proportional to size sampling, where subjects with larger than average ICVs were more likely to be assigned to the large-ICV group and vice versa. The randomization algorithm weighted ICV so that the sex-specific ICV groups had mean differences in ICV that were approximately two standard deviations apart, similar to the mean ICV difference found between men and women (Figure 2).

### ICV-correction methods

#### Proportions method

The oldest, and one of the most commonly applied methods (O'brien et al., 2011) is called the proportions method and is performed by dividing the volume of a neuroanatomical structure by the ICV, thereby creating a ratio, or a “brain fraction” measure (Jernigan et al., 1982). To do this, the raw volumes of each of the 18 structures was divided by the subject's ICV and multiplied by the average ICV for the whole dataset.

#### Residuals method

The residuals method is implemented by running a regression line between the volume of a neuroanatomical structure and ICV, calculated from either a control group or the entire dataset (O'brien et al., 2006). In studies where two healthy groups are compared, such as men and women, the regression line from the entire dataset should be used (Voevodskaya et al., 2014; Nordenskjöld et al., 2015). Similar group sizes and slope of the regression lines (no significant interactions) are essential when regressing across both groups (Nordenskjöld et al., 2015). When interpreting results from the residuals method, uneven group sizes or slopes will introduce bias (Nordenskjöld et al., 2015). From the regression line the “residuals” or difference from predicted volume based on a subject's ICV is calculated (Mathalon et al., 1993). The ICV-corrected measurements are expressed as (Buckner et al., 2004; Raz et al., 2004):

(1) $$Vol_{adj}=Vol-b(ICV-\;\bar{ICV})$$

Here, *Vol_adj_* is the *ICV*-adjusted volume, *Vol* is the original uncorrected volume, *b* is slope from the linear regression of *Vol* on *ICV, ICV* is the ICV for the subject and *ICV* is the mean *ICV* across all subjects.

#### Statistical analyses

All statistical data were analyzed in SPSS 21.0 (IBM Corp., Armonk, NY, USA). Scatterplots of each raw neuroanatomical structure volume vs. ICV were created for men and women combined with a superimposed regression line with corresponding confidence intervals. Independent-sample *t*-tests were run to determine if there were differences in age, ICV, height, and diastolic blood pressure (DBP) between men and women. The participants were stratified in 11 groups according to their ICV, starting from 1000 ml and increasing with 100 ml up to 2100 ml. One-Way ANOVAs were run to determine if the proportion (relative size) of the four different brain tissue types (cortical gray matter, white matter, subcortical gray matter and the ventricles) were different for groups with different ICVs. Two-Way ANOVAs were run to examine the effects of sex and ICV on the relative size of cortical and subcortical gray matter, white matter and the ventricles.

The proportions and residuals methods described above were performed on the ICV-matched subsample and the whole sample. The proportions and residuals methods were also applied to the men- and women-only small vs. large ICV subsamples. To test for significant main effect of sex, as well as sex^*^age and sex^*^ICV interactions, General linear models (GLMs) were used with age, sex and ICV as between subjects factors and neuroanatomical structure as within-subjects factor. Each neuroanatomical structure was tested separately and the significance threshold was set at *p* < 0.05, corrected for multiple comparisons using the Bonferroni-Holm method. These analyses were first performed on the neuroanatomical volumes obtained in the ICV-matched subsample, then the raw volumes and the volumes obtained from the different ICV-correction methods in the entire sample, and finally in the men- and women-only small vs. large ICV subsamples. Subsequently, *post-hoc t*-tests were run to investigate sex differences between the neuroanatomical volumes. The significance threshold was set at *p* < 0.05, corrected for multiple comparisons using the Bonferroni-Holm method. Boxplots of the standardized residuals for all neuroanatomical structures in the ICV-matched subsample and the different ICV-corrected data were created for men and women separately, and in the men- and women-only small vs. large ICV subsamples boxplots were created for the small and large ICV-groups separately. Lastly, to investigate which ICV-correction method had the best match with the ground truth, i.e., the ICV-matched subsample, a Spearman's rank-order correlation was run between the mean standardized volumes for all 18 neuroanatomical structures obtained with the proportions and residuals method and the ICV-matched subsample.

#### Adjusting for additional covariates

Since the ANCOVA method with only ICV as covariate is identical to the residuals method described above, we included additional covariates in the ANCOVA method to investigate their effect on sex differences in neuroanatomical volumes. Additional covariates other than ICV are sometimes included in the statistical regression models in an effort to avoid confounders that affect the outcome of interest (Barnes et al., 2010). The covariates can be included in a single model (ANCOVA) that adjusts the raw volumes for all factors including ICV in one operation. Another approach is to include ICV-corrected volumes in a GLM using additional covariates as predictors.

The impact of other covariates than ICV on sex differences in neuroanatomical volumes was investigated in the ICV-corrected volumes from the proportions and residuals method as well as the volumes from the ICV-matched subsample. To test for significant main effect of sex, as well as interactions between the covariates and sex, age, DBP, height, and sex were included as covariates in GLMs using the ICV-corrected volumes as dependent variable. In addition, ANCOVA analyses were performed which included the uncorrected neuroanatomical volumes as dependent variables and ICV as covariate together with age, DBP, height, and sex (O'brien et al., 2011). These additional covariates were chosen since they are commonly applied and have all been shown to affect the size of different neuroanatomical structures (Swan et al., 1998; Raz et al., 2004; Den Heijer et al., 2005; Ikram et al., 2008; Inano et al., 2013). The values for DBP and height were taken from HUNT3 clinical measurement data, obtained right before HUNT-MRI scanning. Each neuroanatomical structure was tested separately and the significance threshold was set at *p* < 0.05, corrected for multiple comparisons using the Bonferroni-Holm method. Boxplots of the standardized residuals for all neuroanatomical structures were created for men and women separately. Finally, Pearson's product-moment correlations were run to assess the relationship between the covariates and the neuroanatomical structures.

All data are presented as mean ± standard deviation, unless stated otherwise.

## Results

### Participants

**Figure 3:**
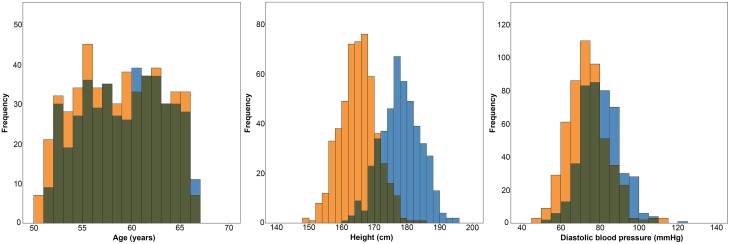
**Histograms showing the distributions of age, height and diastolic blood pressure in women (orange) and men (blue)**.

A total of 1006 subjects (476 males) successfully underwent MRI scanning. 40 individuals were excluded due to motion or image artifacts in the scans (34) and failed FreeSurfer processing (6), leaving 966 (450 males) scans for analysis. ICV was significantly larger in the male group (1666.8 ± 122.9 vs. 1453.6 ± 113.5 ml; *p* < 0.001) (Figure 2). The age range of those included was 50.5–66.8 years, and there was no difference in age between the men and women (58.7 ± 4.1 vs. 58.2 ± 4.3 years; *p* = 0.056). Furthermore, the men were taller (178.1 ± 6.0 vs. 165.1 ± 5.6 cm; *p* < 0.001) and had higher DBP (80.0 ± 10.0 vs. 73.2 ± 10.4 mm Hg; *p* < 0.001) than the women (Figure 3).

### Proportionality between ICV and structural volumes

**Figure 4:**
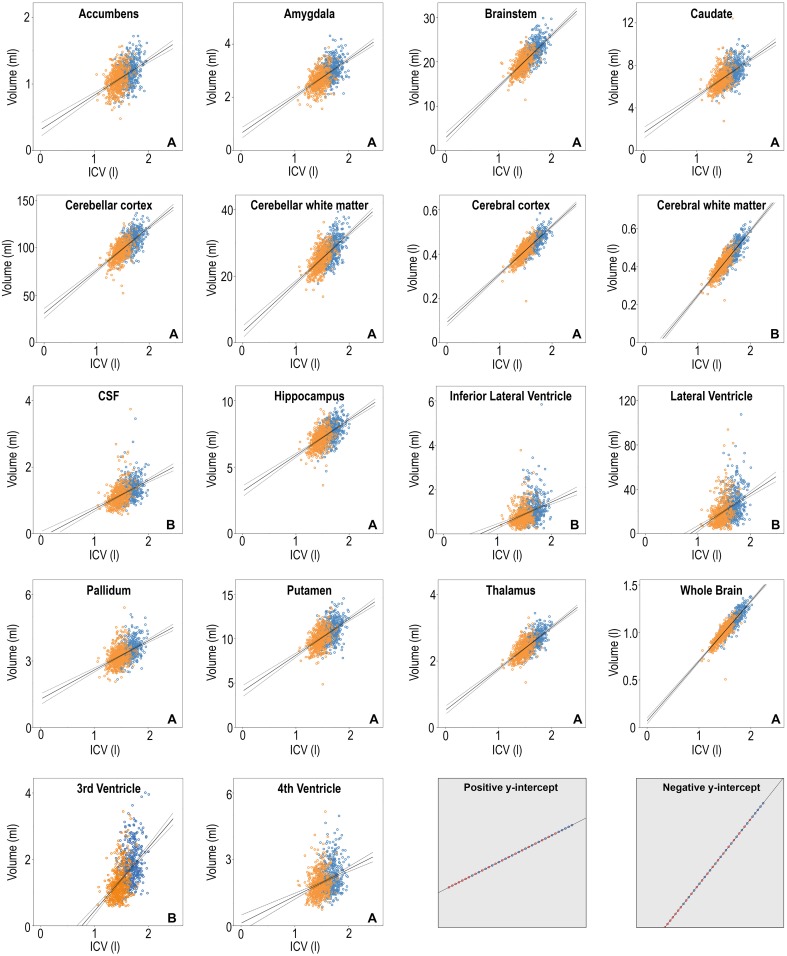
**Scatterplots of the volumes of the 18 neuroanatomical structures vs. ICV, women in orange and men in blue**. The linear regression line for all subjects combined is superimposed in solid line with the 95% confidence interval in dashed. Y-axis: structural volume, x-axis: ICV. The letter A or B in the lower right corner of each neuroantomical volume indicates whether the y-intercept in the regression line is positive or negative. The two gray squares in the lower right corner involve imaginary data. In these cases, a sex difference is clearly not present. The proportions method would however show significant effect of sex because the regression line does not have zero y-intercept.

Figure 4 shows scatterplots of the raw neuroanatomical volumes vs. ICV for men and women. All structures had a linear relationship with ICV and the confidence intervals for the regression lines were generally narrow, with the smaller structures having somewhat wider bands than the larger ones. Although there was a strong correlation between most structures and ICV, no structure was directly proportionate to ICV (i.e., regression line did not go through the origin for any of the volumes). The structures were divided in two groups according to the constant in the regression line; group A had structures with positive y-intercept whereas group B had structures with negative y-intercept. Group A contained all the gray matter volumes (accumbens, amygdala, caudate, hippocampus, pallidum, putamen, thalamus, and the cerebral and cerebellar cortices) whereas group B contained ventricles and white matter structures. In the hypothetical dataset in Figure 4 (shaded squares), women had a relatively larger volume (expressed as proportions of ICV) of the structure with a positive y-intercept, whereas men had a relatively larger volume of the structure with a negative y-intercept. Whether the regression line has a positive or negative y-intercept affects results obtained with the proportions method and will be discussed further below.

### Effects of sex and ICV on the relative size of cortical and subcortical gray matter, white matter, and the ventricles

**Figure 5:**
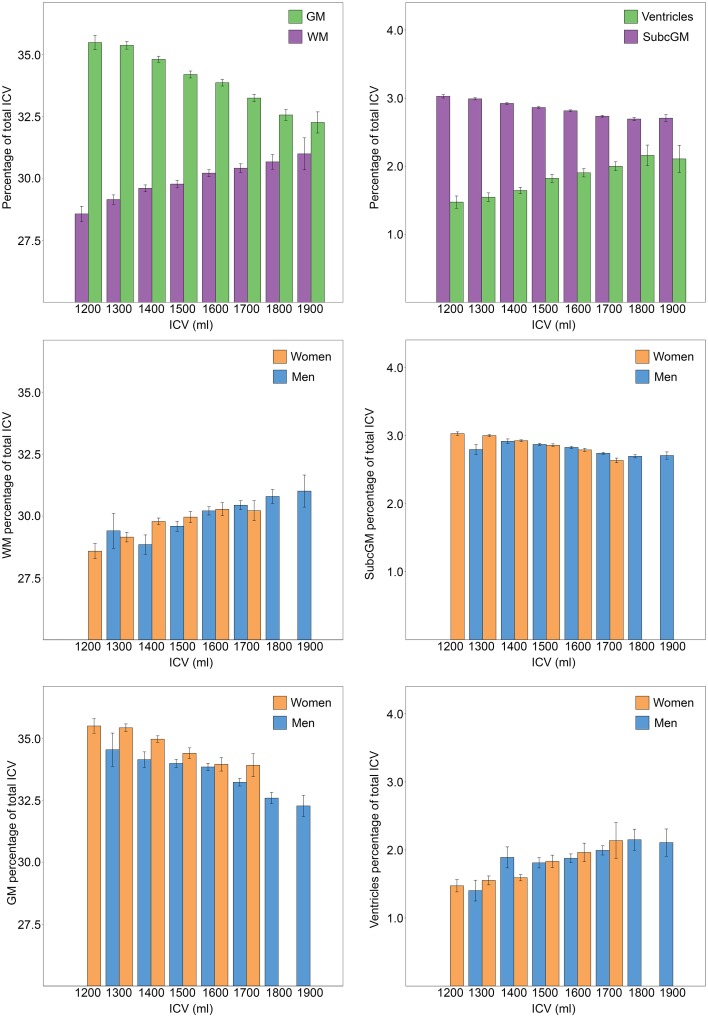
**Relationship between ICV and brain tissue types**. ICV was stratified in groups of 100 ml and groups with less than five participants were excluded. The bars illustrate the relative size (% of ICV) of different brain tissue types with the standard error superimposed. The two top charts are from analysis performed on men and women combined (*n* = 966). A significant effect of ICV was found for all brain tissue types; the relative size of cortical and subcortical gray matter decreased with increasing ICV, the opposite was found for the relative size of white matter and the ventricles. The lower four charts display the relationship between different brain tissue types and ICV for each sex separately. Women had a significantly larger relative size of gray matter. No effect of sex was found for any of the other investigated brain tissue types. GM, gray matter; WM, white matter; SubcGM, subcortical gray matter.

The results from stratifying the ICVs into groups of 100 ml across men and women separately are presented in Table 2 and Figure 5. ICV groups with less than five subjects were excluded. There were no outliers, and there was homogeneity of variances, as assessed by Levene's test. The relative size of cortical [*F*_(7, 952)_ = 26.86, *p* < 0.001, partial η^2^ = 0.165] and subcortical [*F*_(7, 952)_ = 35.99, *p* < 0.001, partial η^2^ = 0.209] gray matter decreased with increasing ICV, the opposite was found for the relative size of white matter [*F*_(7, 952)_ = 8.46, *p* < 0.001, partial η^2^ = 0.059] and the ventricles [*F*_(7, 952)_ = 7.43, *p* < 0.001, partial η^2^ = 0.052].

**Table 2:** **Different brain structures expressed as percentage of ICV**.

**ICV group**	***N***	**Cortical gray matter (% of ICV)**	**White matter (% of ICV)**	**Subcortical gray matter (% of ICV)**	**Ventricles (% of ICV)**
1200–1300 ml	40	35.49 ± 1.85	28.57 ± 1.95	3.03 ± 0.16	1.47 ± 0.57
1300–1400 ml	117	35.38 ± 1.66	29.15 ± 2.04	2.99 ± 0.17	1.54 ± 0.68
1400–1500 ml	222	34.82 ± 1.84	29.61 ± 2.00	2.92 ± 0.18	1.64 ± 0.69
1500–1600 ml	217	34.20 ± 2.06	29.78 ± 2.29	2.86 ± 0.19	1.82 ± 0.88
1600–1700 ml	170	33.87 ± 1.75	30.22 ± 1.84	2.81 ± 0.18	1.90 ± 0.79
1700–1800 ml	135	33.25 ± 1.70	30.42 ± 2.04	2.73 ± 0.15	2.00 ± 0.76
1800–1900 ml	47	32.58 ± 1.55	30.78 ± 1.93	2.69 ± 0.16	2.15 ± 1.06
1900–2000 ml	11	32.27 ± 1.41	31.00 ± 2.14	2.70 ± 0.17	2.11 ± 0.66

*Participants stratified in different groups according to their intracranial volume (ICV). N, number of participants. One-Way ANOVAs were run to determine if the proportion of the structures were different in different ICV-groups. The proportion of cortical and subcortical gray matter decreased with increasing ICV, the opposite was found for the proportion of white matter and the ventricles*.

To determine whether there were sex differences in the relationship between the relative size of cortical and subcortical gray matter, white matter and the ventricles and ICV, Two-Way ANOVAs were run. No significant interactions between ICV and sex were found for any of the investigated tissue types. The analysis of the main effect for sex showed that women had significantly larger relative size of cortical gray matter, [*F*_(1, 946)_ = 5.602, *p* = 0.006, partial η^2^ = 0.006]. There were no statistically significant sex differences in the relative size of subcortical gray matter [*F*_(1, 946)_ = 0.244, *p* = 0.621, partial η^2^ = 0.000], white matter [*F*_(1, 946)_ = 0.413, *p* = 0.521, partial η^2^ = 0.000] or the ventricles [*F*_(1, 946)_ = 0.038, *p* = 0.846, partial η^2^ = 0.000].

### Interaction between age, ICV, and sex in the ICV-matched subsample and entire cohort

There were no significant interactions between age and sex or ICV and sex on any of the 18 investigated neuroanatomical structures. Thus, only results from the *post-hoc t*-tests that compare the volumes between men and women are presented.

### Sex differences in neuroanatomical structures in the ICV-matched subsample

**Figure 6:**
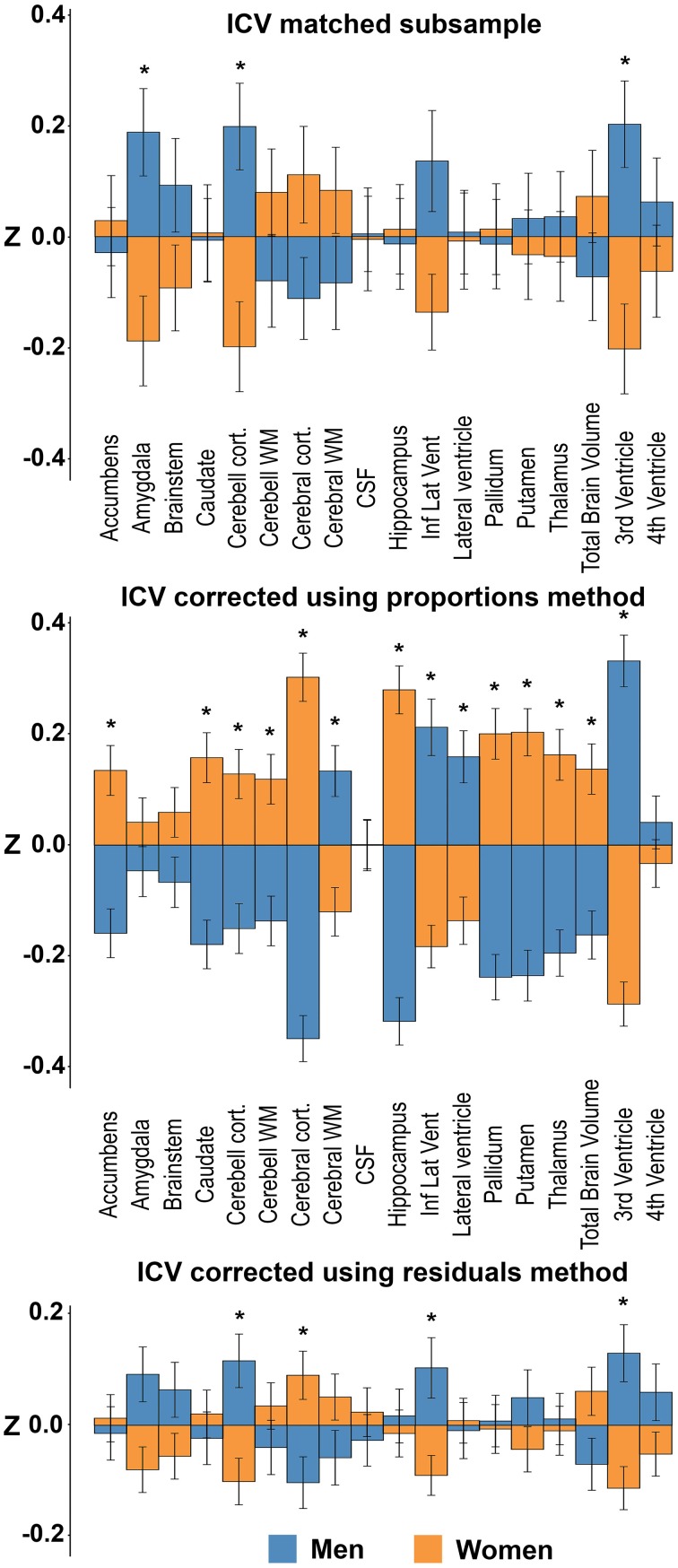
**Sex differences in the ICV-matched subsample and using the different ICV-correction methods**. The bars illustrate mean standardized volume for men (blue) and women (orange) with the standard error superimposed. Y-axis: Z-score of the residuals. **Top**: ICV-matched subsample. **Middle**: ICV-corrected using the proportions method. **Bottom**: ICV-corrected using the residuals method. ^*^Corrected *p* < 0.05.

The subsample consisted of 304 subjects (152 men) with matching ICV (1554.1 ± 87.2 vs. 1554.5 ± 87.0 ml; *p* = 0.97) (Figure 1). There were no differences in age between the two matched groups (men: 58.5 ± 4.4 vs. women: 57.8 ± 4.2 years; *p* = 0.141). The ICV-matched subsample showed that men had significantly larger amygdala, cerebellar cortex and 3rd ventricle, while no structures were significantly larger in women (Table 3 and Figure 6).

**Table 3:** **Volume differences between men and women using different ICV-correction methods**.

**Structure**	**Uncorrected data (*n* = 966)**		**ICV-matched subsample (*n* = 304)**		**Proportions method (*n* = 966)**		**Residuals method (*n* = 966)**	
	**Men**	**Women**	**Men**	**Women**	**Men**	**Women**	**Men**	**Women**
Accumbens	**1.16 (0.18)**	1.06 (0.17)^***^	1.11 (0.18)	1.12 (0.18)	**1.09 (0.16)**	1.13 (0.17)^***^	1.11 (0.17)	1.11 (0.16)
Amygdala	**2.99 (0.37)**	2.63 (0.34)^***^	**2.89 (0.35)**	2.75 (0.36)^*^	2.79 (0.33)	2.82 (0.33)	2.83 (0.34)	2.77 (0.31)
Brainstem	**22.21 (2.28)**	19.56 (1.97)^***^	20.87 (2.04)	20.51 (1.87)	20.71 (1.70)	20.94 (1.80)	20.91 (1.81)	20.70 (1.62)
Caudate	**7.39 (0.93)**	6.71 (0.90)^***^	7.02 (0.89)	7.03 (1.02)	**6.89 (0.78)**	7.18 (0.85)^***^	7.01 (0.82)	7.04 (0.80)
Cerebellar cortex	**107.58 (9.54)**	96.16 (9.19)^***^	**103.60 (8.77)**	99.98 (9.12)^**^	**100.48 (8.57)**	102.98 (9.05)^***^	**102.45 (8.55)**	100.63 (7.97)^*^
Cerebellar white matter	**27.82 (3.53)**	24.93 (3.19)^***^	26.04 (2.99)	26.51 (2.80)	**25.93 (2.72)**	26.66 (2.92)^***^	26.16 (2.96)	26.38 (2.70)
Cerebral cortex	**450.94 (37.61)**	410.06 (37.43)^***^	425.27 (32.82)	433.32 (38.73)	**420.55 (24.25)**	438.47 (27.18)^***^	**426.44 (25.49)**	431.43 (25.44)^*^
Cerebral white matter	**474.27 (54.59)**	405.95 (48.38)^***^	433.37 (46.00)	440.76 (42.40)	**441.29 (31.32)**	433.11 (31.94)^***^	435.97 (32.33)	439.35 (29.17)
CSF	**1.30 (0.29)**	1.14 (0.31)^***^	1.23 (0.27)	1.23 (0.37)	1.22 (0.26)	1.21 (0.29)	1.21 (0.28)	1.22 (0.28)
Hippocampus	**7.67 (0.73)**	7.09 (0.68)^***^	7.35 (0.70)	7.37 (0.69)	**7.17 (0.66)**	7.60 (0.71)^***^	7.37 (0.65)	7.35 (0.61)
Inferior lateral ventricle	**1.12 (0.55)**	0.81 (0.39)^***^	1.06 (0.54)	0.93 (0.41)	**1.04 (0.50)**	0.86 (0.41)^***^	**1.00 (0.54)**	0.91 (0.38)^*^
Lateral ventricles	**25.85 (12.86)**	19.41 (11.19)^***^	23.18 (11.92)	22.98 (13.69)	**23.96 (11.47)**	20.55 (11.18)^***^	22.30 (12.29)	22.51 (10.53)
Pallidum	**3.47 (0.42)**	3.19 (0.40)^***^	3.29 (0.40)	3.30 (0.40)	**3.24 (0.36)**	3.42 (0.43)^***^	3.32 (0.38)	3.32 (0.38)
Putamen	**10.88 (1.15)**	9.94 (1.03)^***^	10.44 (1.09)	10.37 (1.08)	**10.16 (1.07)**	10.65 (1.06)^***^	10.43 (1.08)	10.33 (0.94)
Thalamus	**13.00 (1.24)**	11.67 (1.18)^***^	12.32 (1.26)	12.23 (1.25)	**12.13 (0.91)**	12.49 (1.07)^***^	12.30 (0.96)	12.28 (0.98)
Total brain volume	**1115.09 (94.55)**	986.41 (89.44)^***^	1040.20 (79.70)	1052.06 (83.88)	**1039.09 (45.98)**	1054.02 (51.57)^***^	1042.88 (49.14)	1049.38 (48.85)
3rd ventricle	**1.80 (0.56)**	1.28 (0.45)^***^	**1.65 (0.52)**	1.43 (0.54)^**^	**1.67 (0.49)**	1.36 (0.45)^***^	**1.58 (0.53)**	1.47 (0.43)^**^
4th ventricle	**2.18 (0.65)**	1.86 (0.54)^***^	2.05 (0.58)	1.97 (0.61)	2.04 (0.59)	1.99 (0.57)	2.05 (0.64)	1.98 (0.53)

*T-tests of mean sex differences in the 18 volumes using uncorrected data, the different ICV-correction methods and for the ICV-matched subsample. Results are shown in milliliters, as mean (SD). Significant results are in bold*.

* Corrected p <0.05;

** Corrected p <0.01;

*** *Corrected p <0.001. See Materials and Methods for details on the different ICV-correction methods and the ICV-matched subsample*.

### Sex differences in neuroanatomical structures with and without ICV-correction in the entire sample

Presence or absence of significant sex differences in neuroanatomical structures with no correction and with the different ICV-correction methods are presented in Table 3 and Figure 6. Using the raw volumes with no ICV-correction, all structures were significantly larger in men. Using the proportions method to correct for ICV, men had larger cerebral white matter, inferior lateral ventricle, lateral ventricle and 3rd ventricle (all with a negative y-intercept, group B), whereas women had larger nucleus accumbens, caudate, cerebellar cortex and white matter, cerebral cortex, hippocampus, pallidum, putamen, thalamus, and total brain volume (all with a positive y-intercept, group A). Using the residuals method to correct for ICV, men had larger cerebellar cortex, inferior lateral ventricle and 3rd ventricle, whereas women had larger cerebral cortex.

### Neuroanatomical volumes in the men- and women-only dichotomized into small vs. large ICV subsample

**Figure 7:**
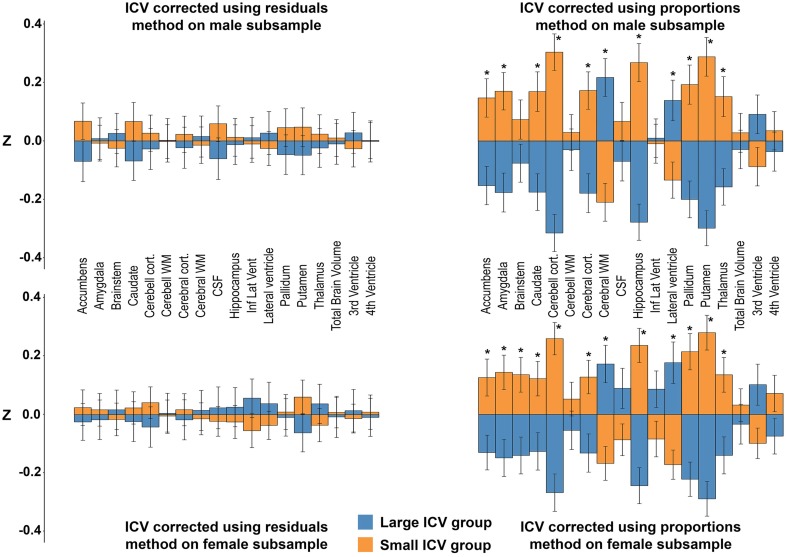
**Differences between the large vs. small ICV-groups in the men- and women-only subsamples**. The bars illustrate mean standardized volume for the large-ICV groups (blue) and small-ICV groups (orange) with the standard error superimposed. Y-axis: Z-score of the residuals. **Top left:** ICV-corrected using the residuals method on the male subsample. **Top right:** ICV-corrected using the proportions method on the male subsample. **Bottom left:** ICV-corrected using the residuals method on the female subsample. **Bottom right:** ICV-corrected using the proportions method on the female subsample. ^*^Corrected *p* < 0.05.

The subsamples consisted of 450 (men) and 516 (women) subjects, respectively. There were no differences in age between the large- and small-ICV groups in the male (59.1 ± 4.0 vs. 58.4 ± 4.2 years; *p* = 0.116) or female (58.2 ± 4.2 vs. 58.2 ± 4.4 years; *p* = 0.882) subsample. Each of the four groups had normally distributed ICVs, and mean ICV was significantly larger in the large-ICV group in the male (1758.6 ± 77.7 vs. 1578.2 ± 88.7 ml; *p* < 0.001) and female (1529.4 ± 77.1 vs. 1380.2 ± 93.0 ml; *p* < 0.001) subsample (Figure 2). Using the residuals method, no structures were significantly different between the small and large ICV groups for either sex. When using the proportions method to correct for ICV in the men-only subsample, the large-ICV group was found to have larger cerebral white matter and lateral ventricle (both with a negative y-intercept, group B), whereas the small-ICV group had larger accumbens, amygdala, caudate, cerebellar, and cerebral cortex, hippocampus, pallidum, putamen, and thalamus (all with a positive y-intercept, group A). Using the proportions method on the women-only subsample gave identical results. In addition, the small-ICV women group had significantly larger brainstem (group A) (Figure 7).

### Correlations between the ICV-correction methods across the entire sample and the ICV-matched subsample

The residuals method was most comparable to the ground truth, with same results with regard to sex differences for 15 out of 18 investigated structures (83%) and had a very strong correlation with the ground truth, *r_s_* = 0.889; *p* < 0.001. No significant correlation between the proportions method and the ground truth were found, and there were comparable results on only 4 out of 18 structures (22%).

### Sex differences in neuroanatomical structures after including other covariates

**Figure 8:**
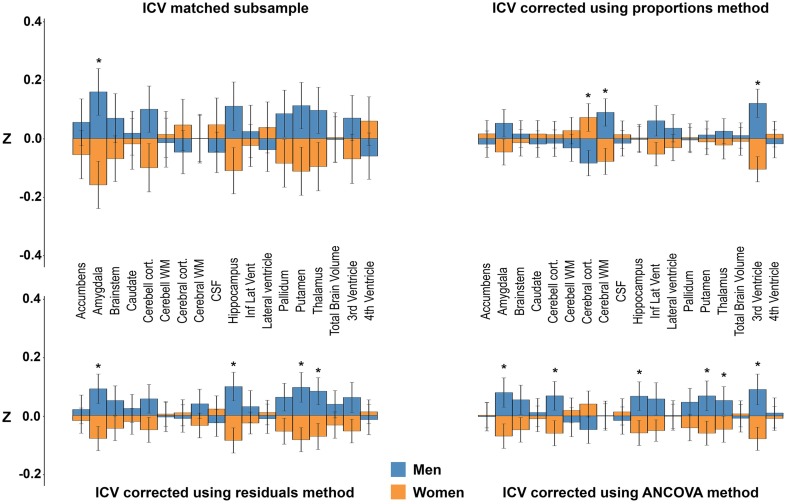
**Sex differences after adjusting for ICV, age, height, and diastolic blood pressure**. The bars illustrate mean standardized volume for men (blue) and women (orange) with the standard error superimposed. Y-axis: Z-score of the residuals. **Top left:** ICV-matched subsample. **Top right:** ICV-corrected using the proportions method. **Bottom left:** ICV-corrected using the residuals method. **Bottom right:** ICV-corrected using the ANCOVA method. ^*^Corrected *p* < 0.05. See Materials and Methods for details on how the covariates were included in the different models.

Age, height, and DBP were included as covariates in GLMs using the volumes from the ICV-matched subsample, and the residuals and proportion methods ICV-corrected volumes as dependent variables. In addition, the uncorrected neuroanatomical volumes were used as dependent variables and ICV as covariate together with age, height, and DBP in a separate ANCOVA analysis (O'brien et al., 2011). Significant results are shown in Figure 8. No significant interactions between the different covariates and sex were found, thus we report only the main effects of sex. In the ICV-matched subsample, men had larger amygdala, *F*_(1, 298)_ = 15.87, *p* = 0.002. With the ICV-proportions method, cerebral white matter, *F*_(1, 952)_ = 15.09, *p* = 0.001, and the 3rd ventricle, *F*_(1, 952)_ = 25.01, *p* < 0.001, were larger in men, whereas cerebral cortex, *F*_(1, 952)_ = 12.01, *p* = 0.002, was larger in women. With the ICV-residuals method, the amygdala, *F*_(1, 952)_ = 17.27, *p* = 0.001, hippocampus, *F*_(1, 952)_ = 20.37, *p* < 0.001, putamen, *F*_(1, 952)_ = 19.25, *p* < 0.001, and thalamus, *F*_(1, 952)_ = 14.24, *p* = 0.003, were larger in men. No structures were larger in women. Finally, in the ANCOVA models where ICV and the other covariates were entered into the same model, and the uncorrected neuroanatomical volumes were the dependent variables, the amygdala, *F*_(1, 951)_ = 22.08, *p* < 0.001, cerebellar cortex, *F*_(1, 951)_ = 13.59, *p* = 0.004, hippocampus, *F*_(1, 951)_ = 16.84, *p* = 0.001, putamen, *F*_(1, 951)_ = 18.34, *p* < 0.001, thalamus, *F*_(1, 951)_ = 11.94, *p* = 0.007, and 3rd ventricle, *F*_(1, 951)_ = 12.74, *p* = 0.005, were larger in men. No structures were larger in women.

There was no interaction between sex and the different covariates (age, height, DBP) on the volumes of the neuroanatomical structures. Still, when examining the effect of the covariates on the volumes of the neuroanatomical structures in men and women separately, both similarities and differences in which structures were affected were present in the two groups. For age, negative correlations were found between all gray and white matter structures apart from caudate and pallidum, and a positive correlation for volume of all ventricles were found in both men and women. Pallidum only correlated with age in women. For height a more complex pattern was present. There were positive correlations between height and the volume of the brainstem, total brain and cerebellar and cerebral white and gray matter in men and women. However, height was also correlated with 4th ventricle, accumbens, and thalamus volumes in men, but not in women. No significant correlations between DBP and the volume of the 18 neuroanatomical structures were found in either sex.

## Discussion

The current study demonstrates sex differences in the volume of some neuroanatomical structures that cannot be explained by ICV in a large, ICV-matched dataset from a representative sample of a general population where participant and non-participant characteristics are described (Honningsvag et al., 2012; Krokstad et al., 2013). Furthermore, the residuals method was shown to be superior to the other ICV-correction methods, and we recommend this method for studies of differences in neuroanatomical volumes. However, it should be noted that including other covariates than ICV in the ANCOVA led to the presence of significant sex differences not present in the ICV-matched sample or in the residuals method (including ANOCVA with only ICV). The results obtained with the proportions method were least similar to the ICV-matched subsample, and the volume differences ascribed to sex differences were related to both ICV and the y-intercept of the regression line between ICV and the neuroanatomical structure. To the best of our knowledge, this is the first study to examine sex differences in neuroanatomical structures in a large age- and ICV-matched sample from a representative general population cohort, and to explore the effect of proportionality with ICV on the same structures.

### ICV-matched subsample

As a ground truth measure of the impact of sex on the volumes of neuroanatomical structures, a large ICV-matched subsample of men and women was created. Since the groups were ICV-matched and there were no differences in age, barring additional confounders, any differences in the neuroanatomical structures in this subsample should be attributable to sex. The current study verified in the ICV-matched subsample, that men have significantly larger volumes of the amygdala, cerebellar cortex and 3rd ventricle compared to women. None of the investigated structures were larger in women. The methods for segmentation of different brain structures are under constant development. Most early studies on sex differences investigated the CSF-filled compartments combined, and several of these earlier publications report larger total ventricular volumes in men (Gur et al., 1999; Chen et al., 2007), consistent with our findings. Further, there are studies to support larger amygdala (Caviness et al., 1996; Goldstein et al., 2001; Fjell et al., 2009; Herting et al., 2014) and cerebellar cortex (Giedd et al., 1996; Raz et al., 2001; Carne et al., 2006; Fjell et al., 2009) in men. The ICV-matched subsample was obtained by matching subjects solely based on their ICV, and therefore only men in the lower ICV range and women in the higher ICV range were included in the final sample. This may introduce selection bias. However, comparing men with relatively small and women with relatively large ICVs should decrease rather than increase sex differences in neuroanatomical volumes if these are proportional to ICV. Thus, any sexual dimorphism in neuroanatomical volumes detected in a sample matched with the current approach should be decreased rather than augmented. In summary, the ICV-matched subsample showed that men have significantly larger volumes of the amygdala, cerebellar cortex and 3rd ventricle than women.

### The residuals method

Four structures were significantly different in men and women after correcting for ICV using the residuals method on the whole sample; larger cerebellar cortex, inferior lateral ventricle and 3rd ventricle in men, larger cerebral cortex in women. These results are in line with data obtained in several studies of sex differences in neuroanatomical structures in both younger and older subjects using the residuals method (Carne et al., 2006; Fjell et al., 2009). The cerebellar cortex and 3rd ventricle were also significantly larger in men in the ICV-matched subsample while the sex differences in the volumes of the cerebral cortex and inferior lateral ventricle did not reach significance level in the ICV-matched subsample. There was, however, a trend toward a larger volume of the cerebral cortex in women (Cohen's *d* = 0.22) and larger volume of the inferior lateral ventricle in men (Cohen's *d* = 0.27) in the ICV-matched subsample. When computing the effect sizes of the residuals corrected results, comparable effects for the cerebral cortex (Cohen's *d* = 0.20) and inferior lateral ventricle (Cohen's *d* = 0.19) as in the ICV-matched subsample were present. The lack of significant group difference between men and women for cerebral cortex and inferior lateral ventricle volume is therefore likely due to the smaller sample size in the ICV-matched subsample and the small effect size of sex on most neuroanatomical volumes. Moreover, although a significant sex difference in the volume of the amygdala was detected in the ICV-matched subsample, this difference did not survive the correction for multiple comparisons (corrected *p* = 0.09, Cohen's *d* = 0.18) in the whole sample after correcting for ICV using the residuals method. The small effect sizes might explain why some studies on healthy subjects report sex differences, including the current (*n* = 966) and Fjell et al. (2009) (*n* = 1143) and other studies such as Voevodskaya et al. (2014) (*n* = 406) not. Further supporting the validity of the residuals method, no structures differed between the large- and small-ICV groups in the men- and women-only subsamples after applying the residuals method. To sum up, the residuals method showed comparable results to the ground truth. Indeed, if taking the smaller sample size of the ICV-matched subsample into consideration, together with the small effect sizes, the findings obtained in the ICV-matched subsample and with the residuals method were highly similar.

### The proportions method

The sex differences between neuroanatomical volumes obtained with the proportions method were profoundly different from the results in the ICV-matched subsample and with the residuals method (Figure 6). The proportions method is based on the assumption that a structure is directly proportionate to ICV, i.e., the regression line goes through the origin (Perlaki et al., 2014). When this assumption fails, the ratio is still expected to be associated with ICV and to retain a correlation with head size (Mathalon et al., 1993). However, as demonstrated in the current work, the correlation with head size differs markedly for different neuroanatomical structures, leading to a bias where structures are systematically mis-assigned to be either larger (group A in Figure 4) or smaller (group B in Figure 4) than their actual size. Indeed, whether there was a positive or negative y-intercept of the regression line (group A or B) predicted the direction of sex difference detected by the proportions method with an accuracy of 100%. These results extend the work of Nordenskjöld et al. (2015). Moreover, this unwanted effect of the proportions method leads to significant sex differences in the volume of a neuroanatomical structure in a hypothetical dataset (Figure 4), although no actual sex differences in volumes are present. In the men- and women-only dichotomized large vs. small ICV subsamples, the proportions method was shown to demonstrate large and significant differences in several structures between the groups, and the differences were similar for the men- and women-only large vs. small ICV subsamples. The hypothetical results derived from group A or B structures and the results from the small vs. large ICV in men and women only subsamples are very similar to the results obtained with the proportions method in the whole dataset, and by Inano et al. (2013). Since any effect of sex is eliminated in the men- and women-only subsamples, these differences are clearly driven by differences related to ICV and not sex, as predicted by Nordenskjöld et al. (2015). To sum up, the lack of proportionality between neuroanatomical volumes and ICV leads to type I errors and detection of sex differences that are not present.

### The effects of ICV

Even though we found sexual dimorphic structures in the ICV-matched subsample, the main finding of the current work is a tight, but not proportionate relationship between ICV and the size of different neuroanatomical structures, regardless of sex. Furthermore, this finding can be generalized to the different brain tissue types. Indeed, the relative size of cortical and subcortical gray matter decreases with increasing ICV, whereas the relative size of white matter and the ventricles increases with increasing ICV. Importantly, apart from women having slightly larger proportion of gray matter than men, a similar relationship was found in both men and women, and there were no interactions between sex and ICV. To summarize, most sex differences in the volume of neuroanatomical structures are related to differences in the scaling of gray and white matter structures with ICV, which is largely independent of sex. However, some differences do remain even after correcting for these scaling issues, but the effect sizes are small and the effects are restricted to a limited number of structures. Although several studies have previously demonstrated that women have proportionately more gray matter than men and that men have proportionately more white matter than women (Schlaepfer et al., 1995; Gur et al., 1999; Allen et al., 2003; Ikram et al., 2008), in light of the current findings, these results are most likely an effect of different ICVs, rather than an effect of sex. One can speculate as to why the relative sizes of different brain tissue types are not constant across the span of different ICVs. To explain the difference in relative gray matter volumes between men and women, it has been hypothesized that individuals with smaller brains compensate by having proportionately larger gray matter volumes to minimize the difference in the total number of brain cells and thus the total computational power (Gur et al., 1999). This compensation mechanism is most likely mostly dependent on brain size, not sex.

### The impact of additional covariates on neuroanatomical volumes

Since the ANCOVA method with only ICV as covariate is identical to the residuals method described above, we included additional covariates in the ANCOVA method to investigate their effect on sex differences in neuroanatomical volumes. The results differed profoundly compared to those obtained without covariates. For instance, using the residuals corrected volumes with inclusion of the additional covariates, none of the previously reported sex differences (larger cerebellar cortex, inferior lateral ventricle and 3rd ventricle in men, larger cerebral cortex in women) were present, instead a sex difference was found in four new neuroanatomical structures (larger amygdala, hippocampus, putamen, and thalamus in men). Interestingly, the results from the ANCOVA method using the uncorrected volumes were similar, but not identical to the results from including the ICV-corrected volumes generated by the residuals method in a GLM using the additional covariates except ICV. Multicollinearity between ICV and height might explain some of the differences. The differences between the results from the ANCOVA method using the uncorrected volumes and the results from including the ICV-corrected volumes generated by the residuals method in a GLM using the additional covariates except ICV were small and included sex differences in cerebellar cortex and 3rd ventricle volumes present in the ANCOVA analysis that were not detected with the residuals method using the additional covariates. Notably, both of these neuroanatomical volumes were different between men and women in the ICV matched subsample. The results from the proportions corrected volumes changed the most after including the additional covariates. Indeed, the results became more similar to those obtained with both the ANCOVA and residuals method with covariates, most likely caused by height acting as a proxy for ICV in the GLM.

There were no statistical interactions between any of the covariates (age, height, DBP) and sex on the volume of the different structures. However, when examining correlations between neuroanatomical volumes and the covariates in men and women separately, it was evident that there were some differences. This was most prominent for height where several structures correlated differently for height in men and women. Multicollinearity as well as actual sex differences related to height, ICV, and neuroanatomical volumes might explain some of the differences obtained with the different statistical models. The results from the ANCOVA and the separate correlation analyses show the potential problem of adding covariates to the analysis of group differences in the volume of neuroanatomical structures, even though the covariates are potentially clinically relevant, and no statistical interaction is present. The differences in the correlations between the neuroanatomical volumes and covariates (height and age) in men and women suggest a possible negative confounding effect of applying such covariates on the results of the presence of sex differences. This demonstrates clearly that inclusion of covariate(s) needs to be carefully considered, and directly related to a scientific hypothesis to avoid confounding the results.

### Limitations

The definition of the ICV-matched subsample as the ground truth is not without limitations, including introducing selection bias as well as random errors of single measurements (Nordenskjöld et al., 2015). Moreover, the ICV-matched subsample is not independent of the whole sample. However, there is no a priori reason to favor either the proportions or residuals method, and using dependent datasets has the advantage of allowing direct comparison between results from the subsamples and the whole sample. Furthermore, we investigated sexual dimorphism in total volume of different neuroanatomical structures. It has been shown that similar volumes of for instance subcortical gray matter structures can be present although the shape of a structure varies (Menke et al., 2014; Persson et al., 2014). Similarly, examining total cerebral cortex volume may obscure regional differences in cortical volumes. Since local volume differences can give rise to differences in cognition (Evensmoen et al., 2013), such differences may play a significant role for sex differences. Finally, by defining the ICV-matched subsample as the ground truth, we are implying that the relative size of a structure is more important than the absolute size. As stated in the introduction, it is generally believed that ICV-corrected neuroanatomical volumes are more valid than absolute volumes in describing structure-function relationships (Sanfilipo et al., 2004). There is, however, some evidence to support a relationship between the overall brain size and cognitive abilities as well, primarily in non-human primates (Reader and Laland, 2002; Hublin and Coqueugniot, 2006; Marino, 2006; Deaner et al., 2007). Indeed, increased brain volume might be accompanied by increased organizational complexity beyond what is expected by the mere difference in volume (Marino, 2006).

## Conclusions

In an ICV-matched subsample of 304 subjects used to define the ground truth for presence of sexual dimorphism in neuroanatomical volumes, we found men to have significantly larger amygdala, cerebellar cortex and 3rd ventricle compared to women. The residuals and ANCOVA (with only ICV as covariate) methods were more effective than the proportions method in removing the effects of ICV. Moreover, most sex differences in neuroanatomical structures were related to differences in the scaling of gray and white matter structures with ICV. The scaling of neuroanatomical volumes with ICV is independent of sex. Still, some sex differences are present even after correcting for ICV scaling, but the effect sizes were small and restricted to a limited number of structures. Thus, ICV differences largely explains sex differences in neuroanatomical volumes, but an added effect of sex is present in descending order for the following structures; 3rd ventricle, cerebellar cortex and the amygdala. Importantly, the proportions method suffered from systematic errors due to lack of proportionality between ICV and neuroanatomical volumes, and no significant correlation between the proportions method and the ground truth were found. Rather than detecting differences related to sex, the proportions method detects differences related to ICV, independent of sex. Based on our findings we would generally advise against using the proportions method since this method is only valid for structures that are directly proportionate to ICV, of which there were none. Inclusion of covariates other than ICV in the statistical models also poses problems, as the results differed widely compared to those obtained without covariates. The results in the current study explain some of considerable variations in the literature on sexual dimorphisms in neuroanatomical volumes. In conclusion, sex plays a minor role in neuroanatomical differences; most differences are related to ICV.

## Author contributions

Data collection: AH. Study concept and design: CP, AH. Analysis and interpretation of data: CP, TH, HE, AH. Drafting of the manuscript: CP, AH. Revising the manuscript critically: CP, TH, HE, AH. All authors discussed the results, commented on the manuscript and approved the final article.

### Conflict of interest statement

The authors declare that the research was conducted in the absence of any commercial or financial relationships that could be construed as a potential conflict of interest.
